# Probing GABA_A_ receptors with inhibitory neurosteroids

**DOI:** 10.1016/j.neuropharm.2018.02.008

**Published:** 2018-07-01

**Authors:** Sandra Seljeset, Damian P. Bright, Philip Thomas, Trevor G. Smart

**Affiliations:** Department of Neuroscience, Physiology & Pharmacology, UCL, Gower Street, London, WC1E 6BT, United Kingdom

**Keywords:** GABA-A receptor, GABA, Neurosteroids, Pregnenolone sulphate, Inhibition

## Abstract

γ-aminobutyric acid type A receptors (GABA_A_Rs) are important components of the central nervous system and they are functionally tasked with controlling neuronal excitability. These receptors are subject to post-translational modification and also to modulation by endogenous regulators, such as the neurosteroids. These modulators can either potentiate or inhibit GABA_A_R function. Whilst the former class of neurosteroids are considered to bind to and act from the transmembrane domain of the receptor, the domains that are important for the inhibitory neurosteroids remain less clear. In this study, we systematically compare a panel of recombinant synaptic-type and extrasynaptic-type GABA_A_Rs expressed in heterologous cell systems for their sensitivity to inhibition by the classic inhibitory neurosteroid, pregnenolone sulphate. Generally, peak GABA current responses were inhibited less compared to steady-state currents, implicating the desensitised state in inhibition. Moreover, pregnenolone sulphate inhibition increased with GABA concentration, but showed minimal voltage dependence. There was no strong dependence of inhibition on receptor subunit composition, the exception being the ρ1 receptor, which is markedly less sensitive. By using competition experiments with pregnenolone sulphate and the GABA channel blocker picrotoxinin, discrete binding sites are proposed. Furthermore, by assessing inhibition using site-directed mutagenesis and receptor chimeras comprising α, β or γ subunits with ρ1 subunits, the receptor transmembrane domains are strongly implicated in mediating inhibition and most likely the binding location for pregnenolone sulphate in GABA_A_Rs.

This article is part of the “Special Issue Dedicated to Norman G. Bowery”.

## Highlights

• A range of GABA_A_ receptor subtypes are inhibited by pregenolone sulphate.
• Peak GABA curents are less sensitive to inhibition than steady-state currents.
• Desensitised state of GABA_A_ receptors most sensitive to neurosteroid inhibition.
• Inhibition increases with GABA concentration, but not strongly voltage-dependent.
• Pregnenolone sulphate binding site located within subunit transmembrane domains.

## 1 Introduction

γ-aminobutyric acid type A receptors (GABA_A_Rs) are key proteins in the brain for maintaining control of neuronal excitation. They are pentamers composed of three types of receptor subunits selected from: α1-6, β1-3, γ1-3, δ, ε, θ and π (Fritschy and Panzanelli, 2014, Sigel and Steinmann, 2012, Smart, 2015). These receptors are predominantly composed of αβγ receptors that reside at inhibitory synapses, and αβγ, αβδ and αβ receptors which are found in extrasynaptic membrane domains (Farrant and Nusser, 2005, Mody, 2001, Moss and Smart, 2001). In the brain, GABA_A_Rs will be subject to modulation by endogenous ligands and pre-eminent amongst these are the neurosteroids (Belelli and Lambert, 2005). Two major classes have been defined; the potentiating neurosteroids exemplified by derivatives of sex and stress hormones, such as allopregnanalone (Allop) and tetrahydro-deoxycorticosterone (THDOC), and the inhibitory neurosteroids exemplified by pregnenolone sulphate (PS) and dihydroepiandrosterone (Belelli and Lambert, 2005, Seljeset et al., 2015). To date, use has been made of expression systems to study recombinant GABA_A_Rs and neuronal cultures which contain an array of GABA_A_Rs (Eisenman et al., 2003, Park-Chung et al., 1999, Shen et al., 2000, Zaman et al., 1992) all in the context of probing the mechanism of action of the inhibitory neurosteroids. Although we have a clearer idea as to where these neurosteroids bind to GABA_A_Rs from recent X-ray crystallography (Laverty et al., 2017, Miller et al., 2017), a systematic functional analysis of inhibitory neurosteroids at different GABA_A_Rs has not yet been achieved.

Previously, the receptor subtype selectivity of pregnenolone sulphate has been partly examined (Rahman et al., 2006, Zaman et al., 1992, Zhu et al., 1996), including the use of C. elegans GABA receptors to determine receptor domains that are important for PS inhibition (Wardell et al., 2006, Twede et al., 2007). However, the profiling of inhibitory neurosteroid sensitivity at the most common mammalian GABA_A_R subtypes thought to exist in the brain is still incomplete.

The main aim of the present study is therefore to systematically study the modulation of various GABA_A_R subtypes by the inhibitory neurosteroid PS using a single expression system, human embryonic kidney 293 cells (HEK cells), coupled to common analytical techniques to characterise PS inhibition. We have used chimeric receptors to probe the essential structural elements of the receptor subunits that contribute towards PS inhibition. These approaches have allowed a direct comparison between the activities of PS at different GABA_A_R subtypes, and provided an indication as to whether modulation is more likely to be important for the activation of synaptic or extrasynaptic GABA_A_Rs.

## 2 Materials and methods

### 2.1 Cell culture

HEK cells were cultured using Dulbecco's modified Eagle medium (DMEM) supplemented with 10% v/v foetal calf serum (FCS), 100 U/ml Penicillin-G and 100 μg/ml streptomycin (Gibco). Cells were incubated at 37 °C in humidified air with 5% CO_2_. When approximately 70–80% confluent, cells were washed with Ca^2+^- and Mg^2+^-free Hank's balanced salt solution (HBSS; Gibco) and harvested using 0.05% w/v trypsin-EDTA (Gibco). Cells were re-suspended in culture medium and centrifuged at 168 × *g* for 2 min (MSE Mistral, 2000 centrifuge). The cell pellet was resuspended in DMEM-based culture medium and re-plated at appropriate dilutions. For electrophysiology, cells were plated onto 22 mm glass coverslips (VWR international) pre-coated with 100 μg/ml poly-l-lysine (Sigma).

### 2.2 Cell transfection

HEK cells were transfected with murine DNA (except GABA ρ1 subunit DNA which was human) and allowed 16–40 h for expression prior to experimentation. A calcium phosphate protocol was used. cDNAs for individual receptor subunits (1 μg for each subunit) were mixed with 340 mM CaCl_2_ (20 μl) and a HEPES-buffered saline (HBS; 24 μL; 50 mM HEPES, 280 mM NaCl and 2.8 mM Na_2_HPO_4_, pH 7.2). Enhanced green fluorescent protein (pEGFP-C1) was included as a marker for transfection. The total amount of DNA did not exceed 4 μg per coverslip. A transfection ratio of 1:1:1:1 was used for heteromeric receptors (*e.g*. α1:β2:γ2L: with pEGFP-C1), whilst for homomeric receptors, a ratio of 2:1 (β3 or ρ1 with pEGFP-C1) was used.

### 2.3 Site-directed mutagenesis

All subunits were expressed in a mammalian pRK5 vector to achieve high levels of expression. Site-directed mutations were made using the QuikChange kit (Stratagene) or the Phusion kit (Thermo Fisher Scientific) with primer sequences as shown in Table 1. DNA was sequenced using the Sanger Sequencing Service (Source Bioscience, Cambridge, UK). Following successful mutagenesis, larger cultures were grown and constructs were eluted for storage in TE buffer (1 μg/μl) using the Plasmid Maxi kit (HiSpeed^®^, Qiagen). Constructs were kept at −20 °C for long-term storage. All chimeras used in this study were prepared as described previously (Gielen et al., 2015).

**Table 1:** Forward and reverse primer sequences used to generate mutant ρ1 cDNA constructs.

Construct	Forward primer sequence (5′-3′)	Reverse primer sequence (5′-3′)
**ρ1**^P294S^	TCCttaggtatcacaacggtgctgacc	gactctggcaggcacggc
**ρ1**^P294V^	GTCttaggtatcacaacggtgctgacc	gactctggcaggcacggc
**ρ1**^V256S^	cagtaccagcaagaactTCCtttggagtgacgactgttc	gaacagtcgtcactccaaaGGAagttcttgctggt-actg

The α1^V256S^ mutation was generated using the QuikChange kit whereas the ρ1^P294S^ and ρ1^P294V^ mutations were made using the Phusion kit. Codons introducing a point mutation are shown in capital letters.

### 2.4 Patch-clamp electrophysiology

Coverslips with transfected HEK cells were mounted onto a recording chamber fixed to a Nikon Eclipse TE300 microscope with differential interference contrast optics. Cells were continuously superfused with Krebs solution containing (mM): 140 NaCl, 4.7 KCl, 1.2 MgCl_2_, 2.52 CaCl_2_, 11 glucose and 5 HEPES, adjusted to pH 7.4 with 1 M NaOH. Patch pipettes (resistance 2.5–4 MΩ) were filled with K^+^-based internal solution containing (mM): 1 MgCl_2_, 120 KCl, 11 EGTA, 10 HEPES, 1 CaCl_2_ and 2 K_2_ATP, adjusted to pH 7.2 with 1 M NaOH. The osmolarity of the internal solutions was measured using a vapour pressure osmometer (Wescor Inc.), and was in the range 300 ± 10 mOsm/l. All recordings were performed at room temperature.

Whole-cell membrane currents were recorded with an Axopatch 200B amplifier (Molecular Devices). HEK cells were voltage-clamped between −20 and −40 mV. Data acquisition was performed with Clampex 10.3 (Molecular Devices). Currents were filtered at 2 kHz and digitised at 20 kHz via a Digidata 1440A (Molecular Devices). The series resistance was monitored and calculated throughout all recordings by measuring the membrane current responses to 10 mV hyperpolarising voltage steps of 50 ms duration at a frequency of 10 Hz. Recorded cells for which the series resistance varied by more than 30% were discarded. The series resistance was typically in the range 4–10 MΩ.

Control responses to GABA were obtained at regular intervals by applying GABA at a high concentration (EC_80-100_) to obtain an estimate of membrane seal stability and any GABA current run-down over time. These responses were used to normalise subsequent responses that were used to compile the GABA concentration-response curves. A U-tube rapid application system was used for drug applications with a solution exchange time of <100 ms. A recovery period of 2–3 min was allowed between each application of drug to allow recovery from desensitisation and to minimise the run-down of currents.

### 2.5 Analysis of currents

The amplitudes of peak and steady-state GABA-activated currents were measured relative to the baseline holding current prior to GABA application using Clampfit (v10.3.1.5) software (Molecular Devices). To generate GABA concentration-response curves, the peak of each GABA response was normalised to the peak response to a saturating concentration of GABA (1 mM, unless otherwise stated) and expressed as a percentage. Similarly, steady-state GABA currents were expressed as a percentage of the steady-state current measured at a given time point during the application of GABA.

For inhibition of GABA currents, the steady-state current was defined as the current measured at 10 s following the start of GABA/drug application. For slowly declining currents, the amplitude was measured at 10 s and this was used as a proxy for the steady-state. GABA and antagonists were co-applied, unless otherwise stated. To study inhibition of a GABA response by PS, an EC_80_ concentration of GABA (the concentration at which 80% of the maximal response is achieved) was co-applied with PS.

Normalised GABA concentration-response curves were fitted using the Hill Equation,

$$I=Imax\left[\frac{{\left[A\right]}^{n}}{EC_{50}^{n}+{\left[A\right]}^{n}}\right]$$

where *I* is the normalised response to GABA, *Imax* is the control maximum response to saturating GABA (100%), *A* is the concentration of applied GABA, *EC*_*50*_ is the concentration of GABA producing 50% of maximal response, and *n* is the Hill coefficient.

Inhibition curves were fitted using an inhibition equation,

$$I=Imax\left(1-\;\frac{{\left[B\right]}^{n}}{IC_{50}^{n}+\;{\left[B\right]}^{n}}\right)$$

where *I* is the normalised GABA response in the presence of an antagonist, *Imax* is the maximal response in the absence of antagonist, *B* is the concentration of antagonist, *n* is the Hill coefficient and the *IC*_*50*_ is the concentration of antagonist producing 50% inhibition of the GABA response. All data were curve fitted using a non-linear least squares algorithm in Origin 6.0 (Microcal).

### 2.6 Drugs

GABA was dissolved in distilled water and stock solutions (1 M) were kept at 4 °C. Pregnenolone sulphate (Sigma) was dissolved in DMSO to a stock concentration of 20 mM and kept at −20 °C. Picrotoxinin (Sigma) was dissolved in DMSO and stored in 100 mM aliquots at −20 °C.

### 2.7 Statistics

The Kolmogorov and Smirnov test was used to check if data were normally distributed. For parametric data, pairwise comparisons were made using Student's t-test. Statistical comparisons between more than 2 groups were undertaken using a one-way analysis of variance (ANOVA) in conjunction with the Tukey post-hoc test. All statistical analyses were performed in GraphPad InStat 3 (GraphPad Software, Inc.). The threshold for statistical significance was set at p < 0.05. Data are reported as mean ± standard error of the mean (SEM).

## 3 Results

### 3.1 Recombinant GABA_A_Rs and pregnenolone sulphate

**Fig. 1:**
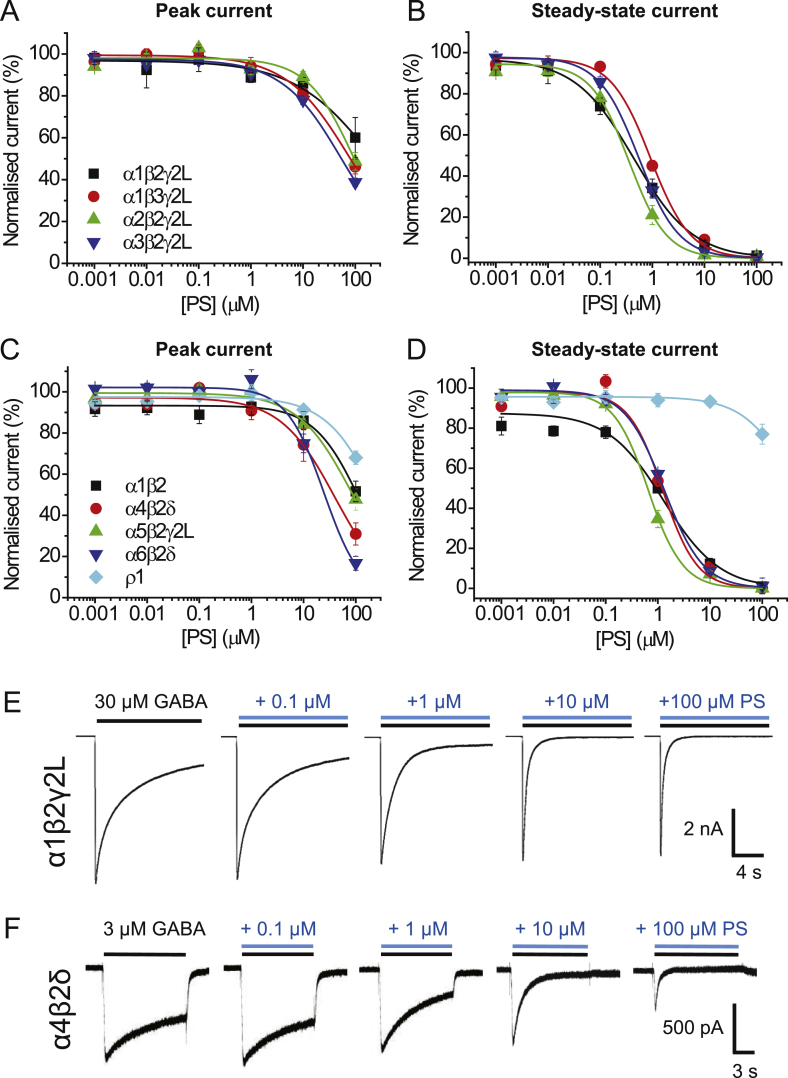
Inhibition of GABA currents by PS at different GABA_A_ receptor subtypes. (A, B) Inhibition of GABA EC_80_ peak (A) and steady-state (B) currents by PS at synaptic-type receptors, including: α1β2γ2L (black), α1β3γ2L (red), α2β2γ2L (green) and α3β2γ2L (blue) (n = 5–7). (C,D) Inhibition of GABA EC_80_ peak (C) and steady-state (D) currents by PS at extrasynaptic-type GABA_A_Rs, including α1β2 (black), α4β2δ (red), α5β2γ2L (green), α6β2δ (blue) and ρ1 (cyan) (n = 5–6). Data points represent mean ± SEM. Curves were fitted as described in the Methods. (E,F) GABA EC_80_ responses and the effect of co-applications with 0.1–100 μM PS at α1β2γ2L, a typical synaptic-type receptor (E), and α4β2δ, a typical extrasynaptic-type receptor (F). (For interpretation of the references to colour in this figure legend, the reader is referred to the Web version of this article.)

To assess the inhibitory activity of PS at GABA_A_Rs, and to determine if the neurosteroid exhibits any receptor subtype selectivity, recombinant receptors incorporating α1-6 subunits with β2 and/or γ2L/δ were systematically expressed in HEK cells and studied using whole-cell electrophysiology. The homomeric ρ1 receptor was also studied as its biophysical and pharmacological profiles are distinct from those of the heteromeric GABA_A_Rs, especially with regard to slower rates of receptor activation and limited desensitisation (Weiss and Chang, 1999), factors which could affect PS inhibition (Seljeset et al., 2015). These receptor subtypes were segregated into those likely to be expressed at inhibitory synapses (synaptic-type, Fig. 1A and B) and those most likely to reside extrasynaptically (extrasynaptic-type; Fig. 1C and D).

The inhibitory effect of PS was observed by co-applying increasing concentrations of PS (0.001–100 μM; Fig. 1) with GABA at an EC_80_ concentration (see Table 2 for GABA concentrations). Under these conditions, PS, only at >10 μM, inhibited GABA peak currents at all the heteromeric receptors examined; however, at <10 μM, PS caused a slowly developing greater block of steady-state currents that increased the apparent rate of desensitisation (Fig. 1E and F).

**Table 2:** GABA_A_ receptor isoform EC_50_s, EC_80_s and Hill slopes (n_H_).

Receptor	GABA EC_50_ (μM)	n_H_	GABA EC_80_ (μM)
α1β2γ2L	4.9 ± 1.4	1.3 ± 0.08	30
α1β3γ2L	7.5 ± 1.2	1.1 ± 0.05	30
α2β2γ2L	21.1 ± 2.1	1.0 ± 0.07	100
α3β2γ2L	64.6 ± 14.3	0.8 ± 0.05	300
α1β2	4.4 ± 0.6	1.0 ± 0.03	20
α4β2δ	0.5 ± 0.08	1.0 ± 0.08	3
α5β2γ2L	10.3 ± 1.5	1.2 ± 0.07	30
α6β2δ	0.2 ± 0.02	1.1 ± 0.08	1
p1	2.6 ± 0.3	1.7 ± 0.1	10

Data were determined from GABA concentration response relationships for each of the listed receptor constructs. All values reported here and used in the text were determined by fitting concentration response curves using the Hill equation outlined in the Methods. Data are accrued from 5 to 7 cells. Values are mean ± SEM.

The IC_50_ for PS inhibition of the steady-state GABA current at α1β2γ2L was 0.4 ± 0.1 μM (n = 7; Fig. 1B). At 100 μM PS, the steady-state current was completely blocked, whilst the peak currents were inhibited by only ∼40%, with little inhibition seen at lower (<1 μM) PS concentrations (Fig. 1A, B, E). These data suggest PS preferentially blocks the steady-state rather than peak GABA currents.

This profile for PS inhibition appeared similar with each receptor subtype studied, except for the ρ1 receptor which was notably less sensitive to PS, with marginal inhibition of the peak and steady-state currents only observed with 100 μM PS (Fig. 1C and D). Otherwise, for the heteromeric αβγ/δ GABA_A_Rs, PS inhibition of steady-state currents yielded IC_50_ values ranging between 0.4 and 1.3 μM (Table 3). For receptors containing β2 and γ2L subunits, expressed with α1, 2, 3 or α5, similar IC_50_ values (p > 0.05) were evident. Replacing β2 with β3 in the α1βγ2L receptor had minimal impact on PS inhibition. In addition, the δ-containing receptors, α4β2δ and α6β2δ, showed comparable sensitivity to PS compared to receptors containing αβ2γ2L subunits, and were similar in terms of IC_50_ to α1β2 (p > 0.05). Together, these results suggest, from the subunits studied, that there is not a strong dependence on the subunit composition for PS inhibition of steady state currents, apart from receptors composed of ρ1 subunits (Table 3).

**Table 3:** Determinations of PS IC_50_s and Hill slopes (n_H_) for GABA_A_ receptor isoforms.

Receptor	PS IC_50_ (μM)	n_H_
α1β2γ2L	0.4 ± 0.05	0.8 ± 0.1
α1β3γ2L	1.0 ± 0.08	1.3 ± 0.2
α2β2γ2L	0.4 ± 0.05	1.3 ± 0.2
α3β2γ2L	0.6 ± 0.05	1.2 ± 0.1
α1β2	1.3 ± 0.07	0.9 ± 0.1
α4β2δ	1.3 ± 0.1	1.4 ± 0.1
α5β2γ2L	0.7 ± 0.1	1.3 ± 0.1
α6β2δ	1.3 ± 0.1	1.1 ± 0.03
p1	>300	–

Data in the table were accrued from PS inhibition concentration relationships for each of the listed receptor constructs. All values were determined from curve fits to the data using the inhibition model outlined in the Methods). Data are from 5 to 8 cells.

By comparison, a differential block by 100 μM PS at various GABA_A_R subtypes became evident when measuring peak currents (Fig. 1A, C). Compared with the inhibition of peak GABA current at α1β2γ2L (60.1 ± 9.6% of GABA control), inhibition by 100 μM PS was significantly increased at α6β2δ (16.7 ± 3.4%; p < 0.001) and at α4β2δ (31 ± 5.4%; p < 0.01). The least sensitive peak currents were those for the ρ1 receptor (68 ± 3.3%) where inhibition was comparable to that observed with synaptic-type α1β2γ2L receptors (p > 0.05).

### 3.2 Access of pregnenolone sulphate to the binding site

The greater inhibition of steady-state over peak currents could arise if the PS binding (association) rate to the GABA_A_R is slow during co-application. This was examined by pre-applying PS for 20 s prior to its co-application with GABA. Inhibition should be increased if slow on-binding is causing the increased steady-state current inhibition.

**Fig. 2:**
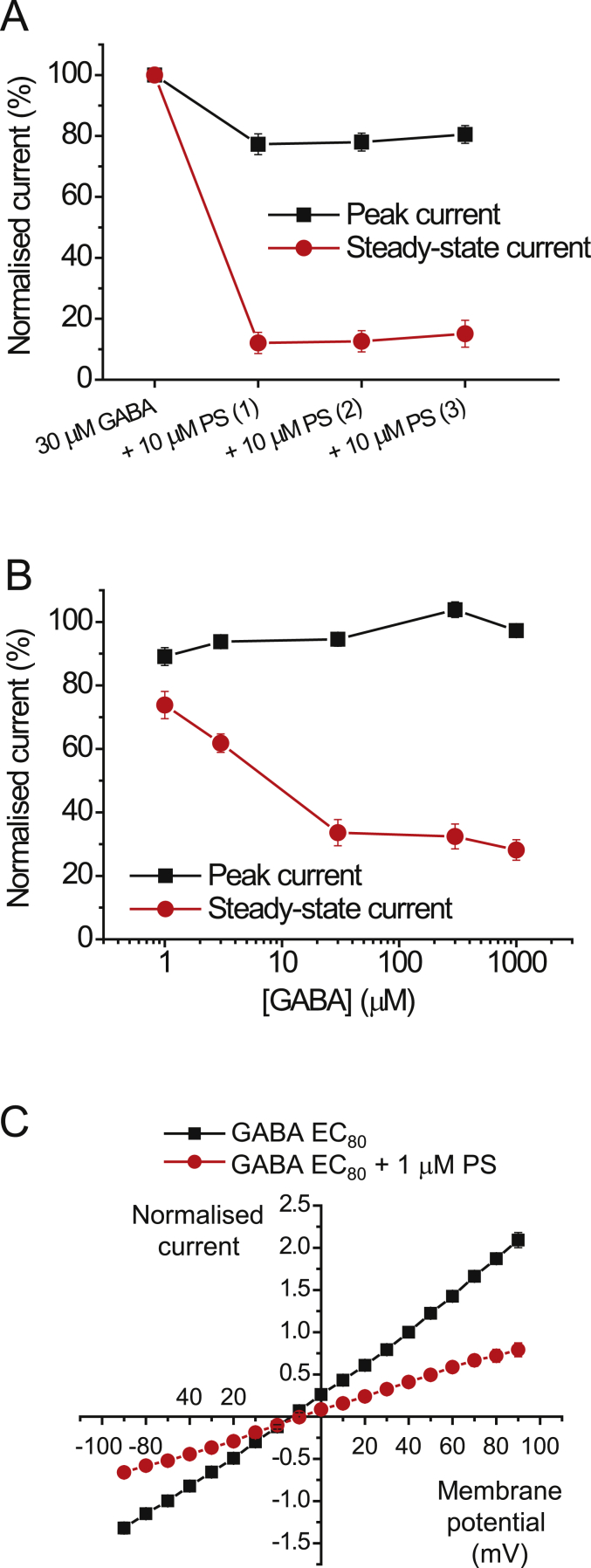
PS inhibition at α1β2γ2L receptors. **(**A) PS inhibition profile showing the peak and steady-state currents for GABA EC_80_ before and after three consecutive co-applications with 10 μM PS at α1β2γ2L receptors (n = 5). PS was not washed out between the three co-applications. (B**)** Inhibition of GABA peak and steady-state currents in response to co-applications of 1 μM PS and increasing concentrations of GABA at α1β2γ2L (n = 7). (C) Current-voltage (I-V) relationships for GABA EC_80_ steady-state currents (black symbols) and when co-applied with 1 μM PS (red) at α1β2γ2L receptors (n = 8). I-V plots were constructed 30 s into a drug application. Currents were normalised to the control GABA response at +40 mV (= 1). (For interpretation of the references to colour in this figure legend, the reader is referred to the Web version of this article.)

PS (10 μM) was pre-applied followed by co-applications with 30 μM GABA (EC_80_) to the α1β2γ2L receptor (Fig. 2A). The steady-state responses to GABA were inhibited, with pre- and then co-applied PS, and remained stable with subsequent co-applications at ∼15% of the GABA control (n = 5, Fig. 2A). Notably, the peak current response remained stable throughout also, and was similar to that observed without pre-application of PS. With pre-application, the peak response to the third application of GABA and 10 μM PS was 80.5 ± 2.9% of control, whereas without pre-application, the response was 85.9 ± 2.8% of control (n = 5, p = 0.2230; Fig. 1, Fig. 2A). These data implied that access for PS to its binding site was unaffected by pre-application and thus unlikely to bind efficiently to the inactive state of the receptor.

### 3.3 GABA_A_R activation and pregnenolone sulphate block

As pre-application of PS did not affect the level of receptor block, we then assessed whether inhibition is receptor state-dependent using α1β2γ2L receptors. PS (1 μM) was co-applied with GABA concentrations from 1 μM (EC_20_) to 1 mM (EC_100_), and peak currents and steady-state currents were measured (Fig. 2B). Whereas inhibition of peak currents by PS was similarly minimal at all concentrations of GABA, a clear increase in steady-state current inhibition was observed with higher concentrations of GABA (n = 7, p < 0.01 comparing 1 and 30 μM GABA), attaining a maximum inhibition at 30 μM GABA (EC_80_). This GABA concentration was used subsequently for experiments regarding PS modulation of GABA_A_Rs.

The observation that PS is a more potent antagonist at higher GABA concentrations could be due to increased open probability of the GABA channel or increased occupancy by GABA, allowing greater access of PS to its binding site. Such a scenario is in accord with a channel or transmembrane domain (TMD) binding site, which implies PS could be a use-dependent blocker. The increase in inhibition observed at high GABA levels may also suggest the block is state-dependent. At higher GABA concentrations a larger proportion of receptors will be desensitised, and PS may only then access its binding site and act as a negative modulator.

To investigate these scenarios, current-voltage (I-V) relationships were first used to determine if antagonism by PS was voltage-sensitive. This behaviour might be expected given that PS has a negatively-charged sulphate group on the C3 position of the A ring. Thus, increased block may develop at depolarised potentials if the binding site ‘experiences’ the membrane electric field. However, the I-V relationship revealed only weak voltage-dependence to the block exerted by 1 μM PS, with inhibition increased by 12% at +90 compared to −90 mV (Fig. 2C; n = 8, p = 0.009). The weak voltage-sensitivity of PS suggests that binding is not strongly affected by the membrane electric field, arguing for a binding site located outside the channel pore or perhaps shielded within the transmembrane domain (TMD). The receptor activation-dependence of PS modulation is therefore more likely to be due to either higher agonist occupancy or a receptor state-dependent block.

**Fig. 3:**
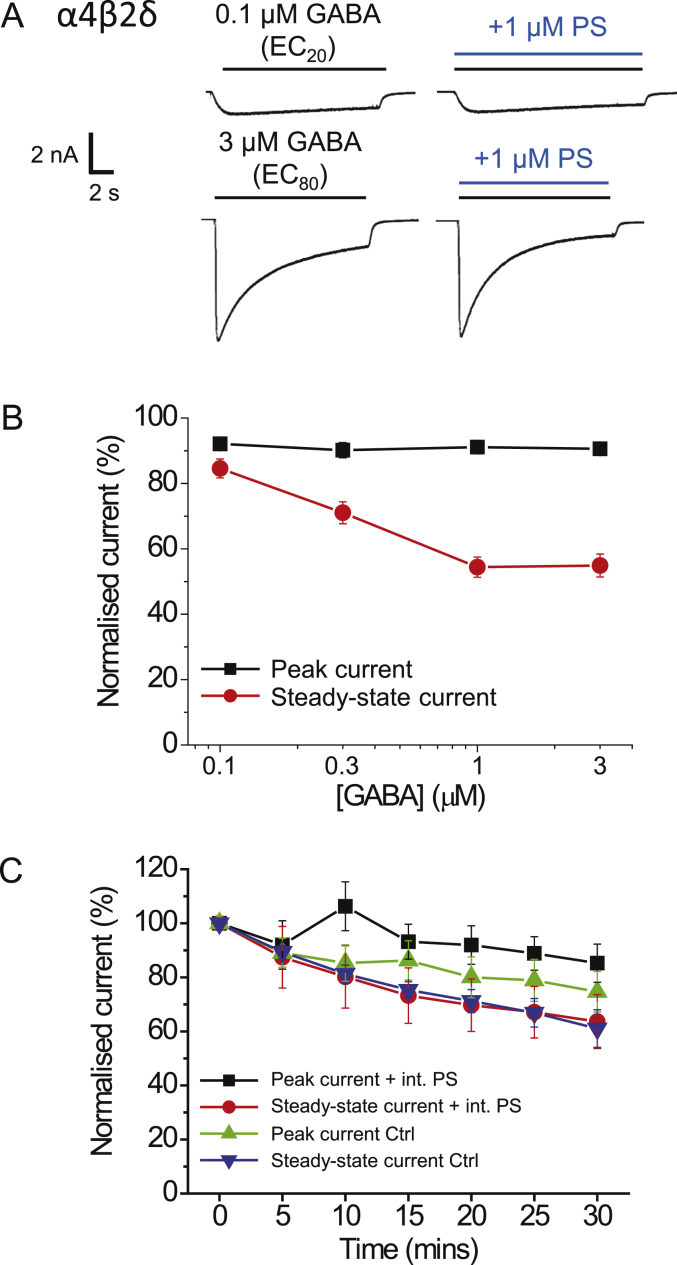
PS inhibition at δ subunit-containing GABA_A_Rs and exclusion of intracellular binding pathway. (A) EC_20_ and EC_80_ GABA-activated currents recorded from α4β2δ receptors show the effect of co-applying 1 μM PS with increasing concentrations of GABA (0.1 or 3 μM) to determine if the inhibition of α4β2δ GABA currents is activation-dependent. (B) GABA concentration profile for PS block of peak and steady-state currents at α4β2δ receptors. PS (1 μM) inhibition was increased by higher GABA concentrations (n = 6). (C) Time profiles for 1 mM peak and steady-state GABA currents at α1β2γ2L receptors applied at 5 min intervals in the absence (Ctrl) or presence (+int. PS) of 100 μM PS applied internally via the patch pipette solution (n = 7–8).

Extrasynaptic GABA_A_Rs will experience markedly reduced GABA concentrations (i.e. nanomolar) compared to those present at inhibitory synapses (millimolar) (Farrant and Nusser, 2005, Glykys and Mody, 2007). To assess whether PS acts as an activation- or state-dependent antagonist at extrasynaptic-type α4β2δ receptors, PS was co-applied with low GABA concentrations from 0.1 μM (EC_15_) to 3 μM (EC_80_) (Fig. 3A). As noted for synaptic-type α1β2γ2L receptors, but now using much lower concentrations of GABA, 1 μM PS exerted greater inhibition as the GABA concentration increased to 1 μM for α4β2δ receptors (Fig. 3B). By contrast, and again in accord with α1β2γ2L receptors, the peak current was minimally affected by PS at all GABA concentrations tested. This demonstrates that PS acts as a state-dependent antagonist also at α4β2δ receptors, suggesting that inhibition by PS is likely to occur at receptors located outside inhibitory synapses where ambient GABA concentrations are likely to be lower.

### 3.4 Internal access for pregnenolone sulphate to its binding site

Clearly PS can access its binding site to inhibit GABA_A_Rs when applied externally. PS has the canonical lipophilic four-ring carbon backbone common to the neurosteroids, and it is derived from cholesterol. Thus, it is possible that PS can partition into the membrane like other neurosteroid molecules (Akk et al., 2009), although this may be affected by the charged sulphate group in ring A. However, whether it can access its binding site from the cytoplasmic side of the membrane like the potentiating neurosteroids (Akk et al., 2005) is unknown. To examine this, 100 μM PS was internally-applied via the patch pipette solution and 1 mM GABA responses were recorded at 5 min intervals for 30 min (Fig. 3C) and compared to control recordings using normal PS-free internal solution. As PS inhibits steady-state currents with minimal effect on the peak current, we expected a larger run-down of the steady-state current in cells with PS-containing internal solution if the neurosteroid can access its binding site from the cytoplasm.

However, the time profiles for the peak and steady-state currents were similar with or without internal solution supplemented with 100 μM PS for up to 30 min (p = 0.324 and 0.833 respectively, n = 8; Fig. 3C). These results imply that PS is unable to inhibit GABA_A_Rs from the cytosolic side of the cell membrane, and suggests that its binding site can only be accessed externally.

### 3.5 Pregnenolone sulphate and the GABA channel

**Fig. 4:**
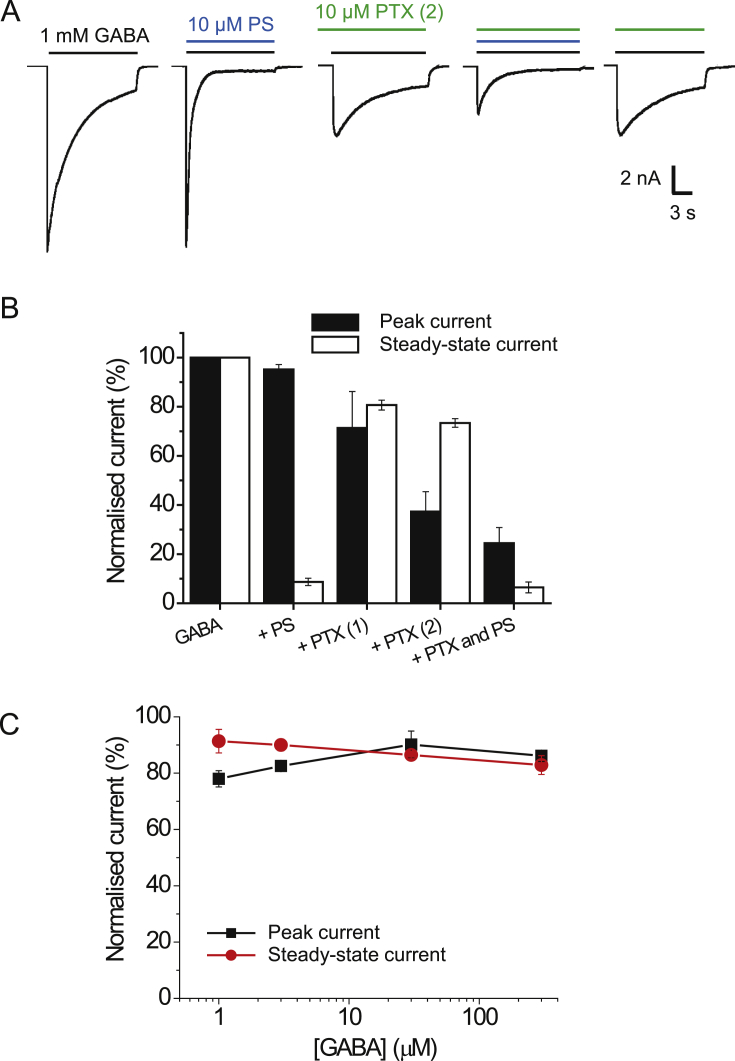
PS and picrotoxin (PTX) do not compete for a common binding site on α1β2γ2L receptors; and activation-dependence of block at ρ1-subunit containing receptors. (A) GABA EC_100_ (1 mM) current responses for α1β2γ2L receptors when GABA is applied alone, and co-applied with 10 μM PS, 10 μM PTX or both. Note PTX was also pre-applied. The response to GABA shown following pre-application of PTX represents the second (2) such response to ensure PTX inhibition is at equilibrium. PTX was continuously kept in the bath from the start of the PTX applications until the end of the experiment. (B) Bar graph showing responses of α1β2γ2L receptors to GABA, PS and PTX (1st and 2nd consecutive responses) as described in (A) (n = 5). Data are expressed as mean ± SEM. (C) GABA concentration profile for PS inhibition of ρ1 homomers using co-application of 30 μM PS with various concentrations of GABA. There is minimal inhibition of either peak or steady-state currents (n = 7).

Given the GABA activation-dependence of PS inhibition, we examined if PS can bind within the GABA channel by employing a competition protocol with picrotoxin (PTX), an antagonist that is considered to operate as an open-channel blocker of GABA_A_Rs and other members of the pentameric ligand-gated ion channel family (Erkkila et al., 2008, Hibbs and Gouaux, 2011, Krishek et al., 1996a). GABA was applied (EC_100_; 1 mM) to obtain stable control responses prior to co-application with 10 μM PS to the α1β2γ2L receptor. After recovery (not shown) PTX (10 μM) was then pre-applied before co-application with GABA. As the PTX block is use-dependent (Yoon et al., 1993), it was applied 2–3 times with GABA to achieve a steady-state inhibition. Finally, GABA and PTX were co-applied with PS to determine if the PTX binding was occluding PS binding and inhibition (Fig. 4A). PS alone had minimal effect on the peak GABA current (95.2 ± 2.0% of control) whilst the steady-state current was greatly reduced (8.7 ± 1.5%; Fig. 4B). By contrast, PTX had a smaller inhibitory effect on the steady-state current (73.4 ± 1.8%, after two applications), but substantially reduced the peak current (37.3 ± 8.1% of control, Fig. 4A and B). When PS and PTX were co-applied, a profound block of both peak and steady-state currents was evident with the peak current reduced to 24.5 ± 6.4% of control and the steady-state current reduced to 6.5 ± 2.2%. Re-applying PTX alone with GABA after wash-out of PS, showed that the inhibition profile in PTX and PS was not due to an enhanced block by PTX (Fig. 4A). The level of block caused by PS was similar in the presence and absence of PTX, indicating that both PTX and PS exert their full inhibitory effect independently when co-applied, thus their binding sites are unlikely to overlap. Combining these data with the low voltage-sensitivity of PS argues for a binding site for the inhibitory neurosteroid located outside the ion channel.

### 3.6 Pregnenolone sulphate and ρ1 homomeric GABA_A_Rs

As the inhibition by PS at ρ1 GABA receptors was distinct and less potent compared to that for other heteromeric GABA_A_R subtypes, we further examined its profile. Given that the extent of PS inhibition at α1β2γ2L depended on the GABA concentration, we investigated whether this also applied to the ρ1 receptor. Co-applying 30 μM PS with GABA at concentrations between 1 μM (EC_20_) and 300 μM (EC_100_) revealed that the peak and steady-state currents were invariant at 80–90% of the GABA control responses (Fig. 4C) suggesting that there is no activation-dependent block at this receptor. Furthermore, pre-application of PS did not increase the level of inhibition of ρ1 receptor-mediated currents (data not shown). These results indicate that ρ1 receptor is only marginally sensitive to PS inhibition and may not contain the molecular signalling pathway necessary for full PS inhibition.

### 3.7 GABA_A_R structural domains sensitive to PS inhibition

**Fig. 5:**
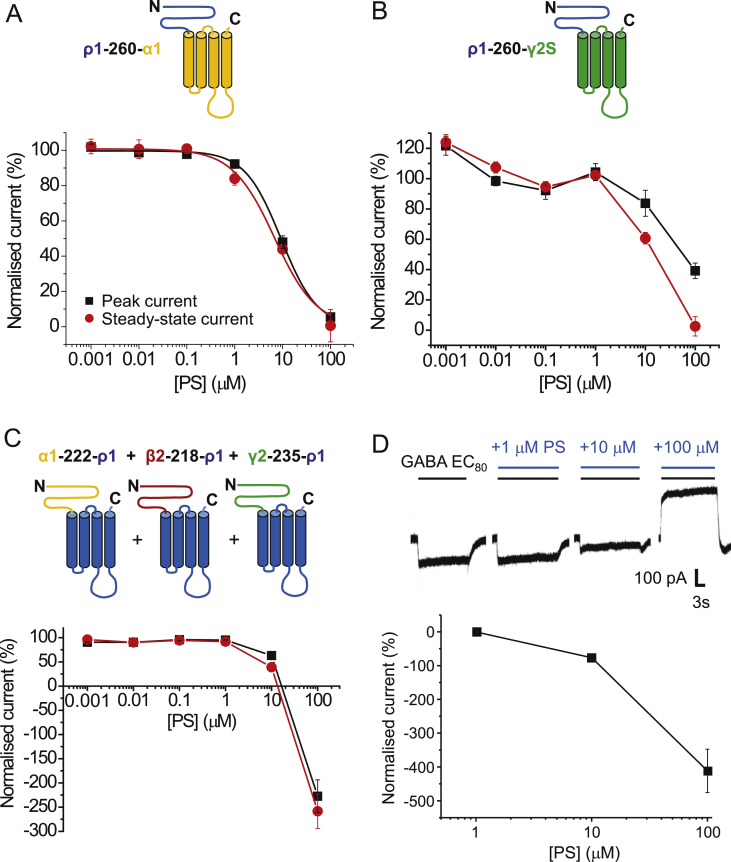
Inhibition by PS at three ECD-TMD GABA_A_R chimeras. (A) Schematic diagram (upper panel) of the transmembrane topology for the ρ1-260-α1 chimera in which the ECD is taken from ρ1 (blue) and the TMD and linkers are from α1 (orange). The lower panel shows PS inhibition data for GABA EC_80_ (30 μM) peak and steady-state currents (n = 5). (B) Schematic (upper panel) for ρ1-260-γ2S chimera in which the ECD from ρ1 (blue) is fused to the TMD from γ2S (green). Lower panel shows PS inhibition data for GABA EC_80_ (200 μM) peak and steady-state currents (n = 5). (C) Upper panel depicts a heteromeric chimera composed of: α1-222-ρ1 + β2-218-ρ1 + γ2-235-ρ1 subunits. ECDs are from α1 (orange), β2 (dark red) and γ2 (green), and the TMD is from ρ1 (blue) for all chimeras. Lower panel shows PS inhibition data for GABA EC_80_ (1 μM) peak and steady-state currents (n = 9). The negative normalised current describes inhibition by 100 μM PS of the constitutive channel activity. Data are expressed as mean ± SEM. (D) Upper panel shows examples of PS inhibition of GABA-activated (inward) and constitutively-active (outward) currents for the heteromeric α1-222-ρ1 + β2-218-ρ1 + γ2-235-ρ1 chimeric receptor. Lower panel shows PS inhibition concentration data for the constitutively-active current.

Given the low sensitivity of ρ1 receptors to PS inhibition, we used this receptor as a null protein to explore receptor domains that are necessary for supporting PS inhibition. To do this we constructed receptor chimeras formed between ρ1 and α1, β2 or γ2 subunits. Our aim was to switch the receptor sensitivities to PS inhibition depending on the heteromeric receptor assembly. The first chimera studied, ρ1-260-α1, contained the complete extracellular domain (ECD) of ρ1 up to residue 260, (i.e. the start of M1, numbered as in the mature protein), with the TMD and its associated linkers taken from the α1 subunit (Fig. 5A). This chimera, designed to determine whether PS is dependent upon the ECD or TMD of α1, was inhibited by PS (Fig. 5A). Although the potency was reduced compared to inhibition at heteromeric wild-type receptors (IC_50_ range 0.4–1.3 μM for steady-state currents), prominent and similar inhibition of both peak and steady-state GABA currents was still observed with IC_50_s of 9.5 ± 1.1 μM and 7.9 ± 1.7 μM, respectively. Virtually full inhibition of the GABA peak and steady-state current was obtained at 100 μM PS. These results show that PS can antagonise a chimera where the ECD is from the largely PS-insensitive ρ1 subunit, and suggested that the neurosteroid is likely to rely on the TMD of α1 for inhibition.

By substituting α1 for γ2S in another chimera, ρ1-260-γ2S, inhibition was again observed at concentrations of PS higher than 1 μM, and full inhibition of the steady-state current was attained at 100 μM PS (Fig. 5B). The peak current was also inhibited by PS, and reached 39.2 ± 5.1% of control at 100 μM PS. These results also imply that the TMD is important for PS inhibition, but for the γ2 subunit-containing chimera, PS was less potent when compared to the potency determined at the ρ1-260-α1 chimera.

Next we investigated a heteromeric receptor chimera using three different chimeric subunits composed of the ECD from α1, β2 and γ2 separately fused to the TMD of ρ1 with its associated linkers (Fig. 5C). Hypothetically, we considered that PS should require the TMD of α1 and γ2 and quite possibly β2, for full inhibition. So with this chimera PS inhibition was expected to be disrupted. However, PS was still able to inhibit the small GABA-activated peak and steady-state currents, though only at 10 and 100 μM (Fig. 5C). Notably, a much larger standing current, caused by constitutive channel activity of the heteromeric chimera, was also revealed following inhibition by PS in the absence of GABA (Fig. 5D). It is likely that PS mostly mediated block of this spontaneous current rather than the GABA-mediated current, given that the outward current was 2–3 fold greater when 100 μM PS was applied in the absence of GABA.

**Fig. 6:**
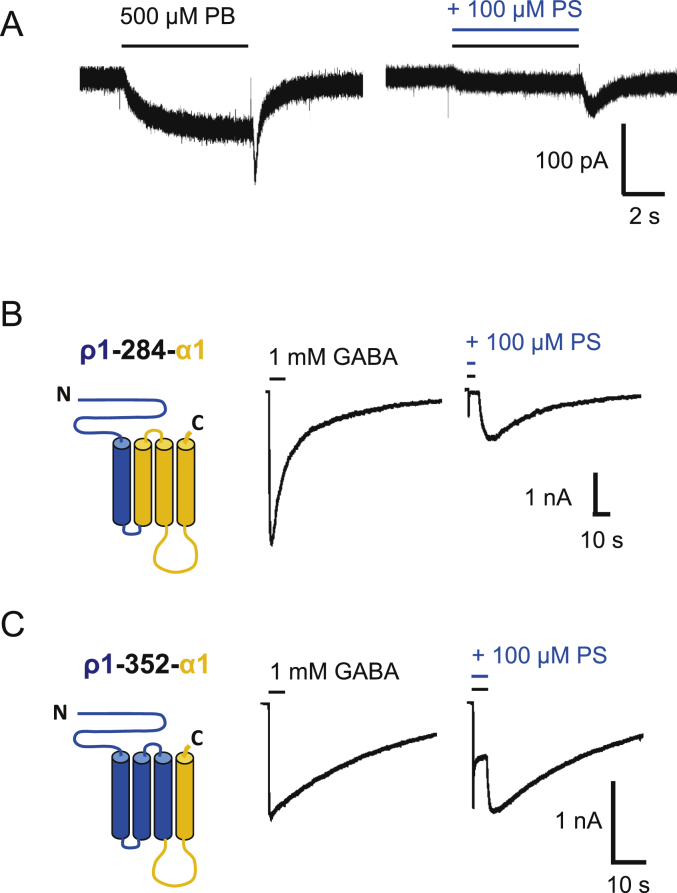
PS inhibition of pentobarbitone (PB) currents at β3 homomers and of GABA currents at TMD chimeras. (A) Membrane currents activated by 500 μM PB at the β3 homomer before (left) and after co-application of 100 μM PS. Note the fast inward rebound current after wash-off of PB and its inhibition by PS. (B) Left panel shows schematic of the transmembrane topology for the ρ1-284-α1 chimera. The ECD, M1 and M1-M2 linker are from the ρ1 subunit (blue), whilst the rest of the chimera is taken from α1 (orange). Right panel shows representative 1 mM GABA currents before and after co-application of 100 μM PS. (C) Left panel shows schematic of the ρ1-352-α1 chimera. The ECD and TMD residues up to the C-terminal end of M3 are taken from ρ1 (blue), whereas the M3-M4 linker and M4 and C-terminal tail are from α1. Right panel shows 1 mM GABA currents and their inhibition by 100 μM PS. At least three independent recordings were performed for each chimera. Note the profound desensitisation and rebound currents.

The ρ1-260-β2 chimera did not functionally express in HEK cells, and thus PS block at this chimera was not determined. Nevertheless, wild-type β3 subunits will form homomeric receptors providing currents gated by pentobarbitone (PB) but not by GABA (Davies et al., 1997, Krishek et al., 1996b, Wooltorton et al., 1997). These currents are slowly activating and followed by a rebound current after agonist wash-off (Wooltorton et al., 1997). To determine if the β3 homomers were subject to inhibition by PS, PB (500 μM) was used as an agonist, and co-applied with 100 μM PS (Fig. 6A). The neurosteroid caused a concentration-dependent block of the PB-induced peak current, and also reduced (though to a lesser extent) the magnitude of the rebound current. This observation argues that a binding site for PS must exist on the β3 subunit. Combined with the data from the chimera studies, these findings suggest that the neurosteroid has the potential to bind to more than one type of subunit of the GABA_A_R (i.e. α, β, γ and ρ1), but most likely this binding involves the TMD.

To probe the TMD of the α1 subunit in more detail for PS inhibition, we created two further chimeras, ρ1-284-α1 (Fig. 6B; ρ1 sequence up to the end of M1), and ρ1-352-α1 (Fig. 6C; ρ1 to the start of the M3-M4 linker). Depending upon the retention or otherwise of PS inhibition with these chimeras, this would indicate the relative importance of discrete areas of the TMD for this inhibitory neurosteroid. GABA-activated currents were all inhibited by 100 μM PS co-applied with 1 mM GABA for both chimeras. The level of peak and steady-state inhibition was greater for the chimera retaining M2-M4, but also retained for the chimera containing M4 alone. These results broadly implicate α1 subunit TMDs as a critical region in PS inhibition, with binding and transduction of the PS inhibitory effect requiring synergy between M2-4.

### 3.8 Inhibition of GABA_A_Rs by PS is affected by the 2’ residue in the ion channel

Although the GABA_A_R subunit TMD is a key region for PS inhibition, previous work has suggested an important role for the 2′ residue near the cytoplasmic end of M2 in recombinant α1β2γ2L receptors. Substituting the 2’ valine in α1 (V256) and the homologous alanine in β2 (A252) for a serine residue caused the association rate for PS inhibition to reduce by 30-fold for α1^V256S^β2γ2L but not α1β2^A252S^γ2L (Akk et al., 2001). Thus the α1 subunit seems important for PS inhibition. Despite these findings, the inhibitory effects of PS are reduced or abolished for *Xenopus* oocytes expressing α1β2^A252S^γ2L or α1^V256S^β2γ2L (Wang et al., 2006, 2007), showing that the mutation in either the α1 or β2 subunit can affect PS sensitivity.

**Fig. 7:**
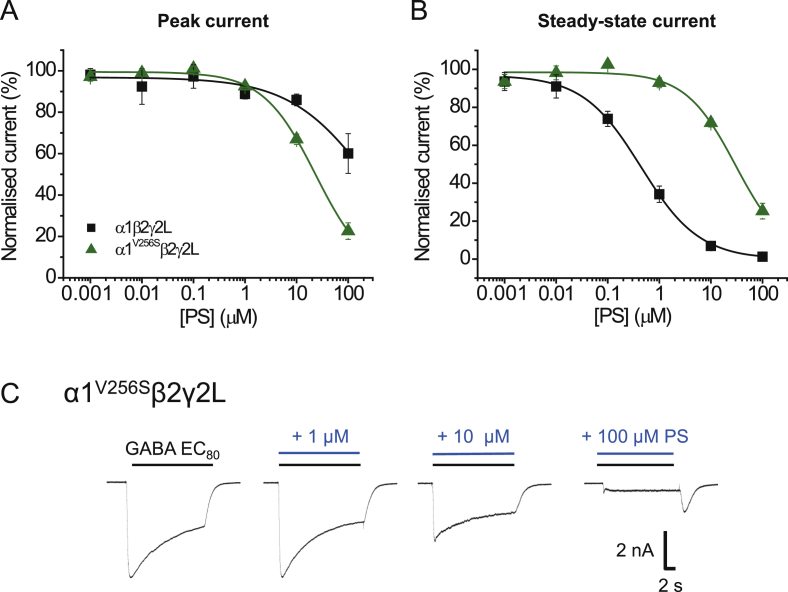
GABA_A_R ion channel 2′ mutant and PS inhibition. (A, B) Inhibition plots for GABA EC_80_ peak (A) and steady-state (B) currents inhibited by PS at α1β2γ2L (black) and α1^V256S^β2γ2L (green) receptors (n = 7–8). Data are presented as mean ± SEM. (C) Representative membrane currents for PS inhibition of GABA-activated currents at α1^V256S^β2γ2L receptors. (For interpretation of the references to colour in this figure legend, the reader is referred to the Web version of this article.)

We examined the importance of the 2’ residue for PS inhibition by examining its role in both the α1 and ρ1 subunits. Initially we recorded GABA whole-cell currents in HEK cells expressing α1^V256S^, β2 and γ2L to assess whether the mutation affected GABA potency and gating of the receptor. For the α1^V256S^β2γ2L receptor GABA was ∼6-fold more potent compared to wild-type, with the EC_50_ reduced from 4.9 ± 1.4 μM (wild-type) to 0.8 ± 0.2 μM for the mutant (p = 0.0104, n = 5–6). We assessed the PS-sensitivity of α1^V256S^β2γ2L in response to GABA EC_80_ application. Although this mutation has been previously reported to reduce or ablate PS sensitivity at α1^V256S^β2γ2L, inhibition was still evident at higher PS concentrations (Fig. 7A and B). However, the curve for PS inhibition of steady-state currents was shifted to the right (0.4 ± 0.1 μM for wild-type α1β2γ2L compared to 35.5 ± 8.2 μM for α1^V256S^β2γ2L; p = 0.0005, n = 7; Fig. 7B), and a greater level of inhibition of the peak GABA current was observed for the α1^V256S^β2γ2L receptor (60.1 ± 9.6% of control for wild-type; 22.6 ± 4.0% of control for α1^V256S^β2γ2L, p = 0.0014, n = 6–7). A rebound current was also present upon wash-off of 100 μM PS, suggesting the channels re-entered an open state before closure (Fig. 7C). Thus, the α1^V256S^β2γ2L mutation shifts the steady-state PS inhibition curve to the right (lower potency), but simultaneously GABA peak currents become more susceptible to inhibition by PS.

As homomeric receptors formed of ρ1 subunits exhibited significantly reduced sensitivity to PS inhibition compared to synaptic and extrasynaptic heteromeric GABA_A_Rs (Fig. 1C and D), we investigated whether the 2′ residue also played a role in modulation by PS at this homomeric receptor, since it is possible that ρ1 lacks the transduction machinery to couple PS binding to receptor inhibition. We therefore switched the ρ1 proline at 2′ for its equivalent valine found in wild-type α1 subunit to assess if this conferred increased sensitivity to PS. We also substituted the 2′ proline for a serine (ρ1^P294S^) as a control since this residue reduced PS potency at the α1β2γ2L receptor. Both mutations shifted the GABA concentration-response curve for ρ1 homomers to the right with the estimated EC_50_ for wild-type ρ1 (2.6 ± 0.3 μM) increased by ∼10-fold for ρ1^P294V^ (24.2 ± 1.1 μM, p < 0.0001, n = 4–8) and ∼3-fold for ρ1^P294S^ (9.7 ± 1.2 μM, p = 0.0009, n = 6–8). By virtue of these curve shifts, the 2’ mutations are likely to affect GABA potency and/or possibly gating kinetics for ρ1.

**Fig. 8:**
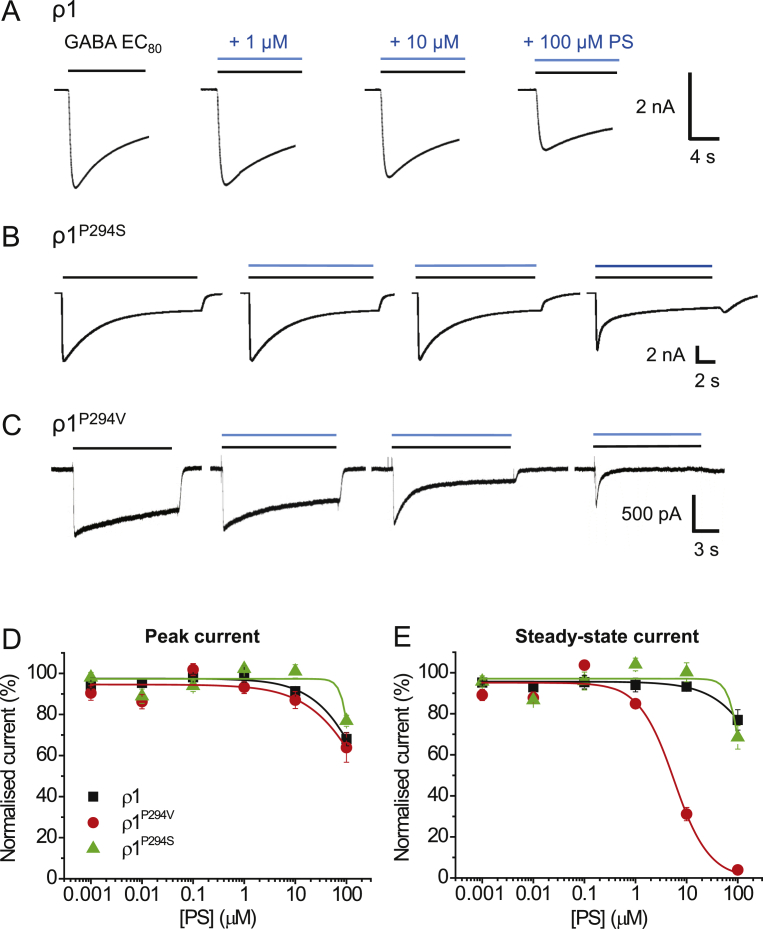
PS inhibition at wild-type and mutant ρ1 receptors. Representative membrane currents for wild-type ρ1 (A), ρ1^P294S^ (B) and ρ1^P294V^ (C) in response to GABA EC_80_ and following co-application with 1, 10 and 100 μM PS. (D, E) Inhibition by PS of GABA-mediated peak (D) and steady-state (E) currents at wild-type ρ1 (black), ρ1^P294V^ (red) and ρ1^P294S^ receptors (green) (n = 6–12). Data are presented as mean ± SEM. (For interpretation of the references to colour in this figure legend, the reader is referred to the Web version of this article.)

To assess the PS sensitivity of the wild-type and mutant ρ1 receptors, EC_80_ GABA was co-applied with PS at increasing concentrations (Fig. 8A–C). The ρ1^P294S^ behaved similarly to wild-type ρ1, with no greater inhibition of the peak or steady-state currents observed (Fig. 8 D, E). By contrast, ρ1^P294V^ was more sensitive to PS, with clear inhibition of the steady-state current observed with PS concentrations higher than 1 μM (Fig. 8C, E). The IC_50_ for steady-state current inhibition by PS at ρ1^P294V^ was 6.0 ± 0.6 μM, approximately 6-fold higher than for wild-type heteromeric αβγ/δ receptors. This result supports a role for the 2’ valine in M2 for signal transduction of PS inhibition at GABA_A_ receptors.

## 4 Discussion

Understanding the mechanism(s) by which inhibitory neurosteroids modulate GABA_A_Rs is important for predicting how this modulation will affect inhibition and ultimately neuronal behaviour in the brain. In addition, knowing whether an endogenous molecule exhibits GABA_A_R subtype selectivity is important for predicting in which regions of the brain and subcellular locations the compound is likely to have most effect. By examining a range of synaptic- and extrasynaptic-type GABA_A_Rs composed of αβγ, αβδ and αβ, the PS IC_50_ for inhibition of the steady-state currents was between 0.4 and 1.3 μM. Although the differences in PS potency at some of the receptor subtypes are statistically different, the small difference (less than 5-fold) suggests that under physiological conditions, subtype selectivity is unlikely for PS inhibition. Nevertheless, a higher efficacy PS block was noted for peak currents at δ-containing receptors, and might imply a greater role for PS in modulating GABA-mediated tonic currents.

The potency of PS is reported to be similar at α1β2γ2L and α5β2γ2L receptors expressed in *Xenopus* oocytes when co-applied with EC_80_ GABA (Rahman et al., 2006), which supports the findings presented here. Furthermore, the potency and efficacy of PS were increased in the presence of the potentiating neurosteroid THDOC (Rahman et al., 2006), which binds to a separate site to PS (Akk et al., 2008, Laverty et al., 2017). This corroborates the hypothesis that high levels of receptor activation or increased channel open probability promote inhibition by PS.

However, contrasting results have also been reported, with α3β2γ2 receptors noted to be 10-fold more sensitive to PS inhibition than α1β2γ2. Moreover, PS was more potent at α6β3γ2 than at α6β3δ receptors suggesting PS potency is reduced by the δ subunit (Zhu et al 1996). This distinction was also noted with α4β3γ2 and α4β3δ, suggesting the γ2 subunit increased the sensitivity to PS (Brown et al., 2002). By contrast, the human ρ1 receptor was notably less sensitive to PS than the heteromeric GABA_A_Rs (Li et al., 2007).

Similarly to the inhibitory neurosteroids, the α subunit does not influence the potency of the potentiating neurosteroids, when co-expressed with β1 and γ2L subunits (Belelli et al., 2002). The β subunit isoform also did not affect allopregnanolone modulation (Belelli et al., 2002, Hadingham et al., 1993), contrasting with PS, where replacement of β2 with β3 in α1βγ2L receptors reduced inhibitory potency. The efficacy of the potentiating neurosteroids does vary between receptor subtypes, with neurosteroid potentiation (macroscopic efficacy) increased at δ-compared to γ2-containing GABA_A_Rs (Akk et al., 2007, Belelli et al., 2002, Wohlfarth et al., 2002).

With regard to GABA occupancy of the receptor, PS inhibition was greater at higher GABA concentrations, which suggested potential activation- or use-dependent block. Inhibition also developed slowly, leading to greater block of steady-state currents than peak currents. Furthermore, applying PS before GABA to the receptors did not increase inhibition, indicating that the slowly developing block is not due to a slow forward rate constant (association) for PS binding to the receptors. Similarly slow kinetics for PS block have been noted for rat hippocampal neurons (Eisenman et al., 2003). These results are consistent with the idea that receptors have to be activated before PS can modulate the response. However, for α1β2γ2L receptors expressed in oocytes, greater inhibition of GABA currents has been observed following PS pre-application (Zaman et al., 1992).

Use-dependent block by PS was discounted since repeated GABA applications in the presence of PS did not further increase inhibition. Thus, the greater block observed at high GABA concentrations is more likely due to a state-dependent block, raising the question as to whether this is due to high receptor occupancy or to a conformation of the receptor caused by high activation levels, *e.g*. receptor desensitisation. A previous study assessed PS inhibitory potency in the presence of a saturating concentration of a GABA_A_R partial agonist, piperidine-4-sulphonic acid (P4S), which attains 30–40% of the maximal GABA response (Eisenman et al., 2003, Mortensen et al., 2004). At this maximum concentration of P4S, the potency of PS was more than 3-fold lower compared to the inhibition of a maximal GABA response. When comparing the fractional block by PS of currents produced by functionally-equivalent concentrations of GABA and P4S, the inhibition by PS of α1β2γ2L receptors expressed in oocytes was similar. This showed that the level of receptor activity, rather than the extent of agonist occupancy, is likely to be a key determinant of PS potency.

The concept of activation-dependent block can be indicative of the antagonist requiring receptor activation to access its binding site, e.g., in the channel pore if located past the channel gate at 9’. For many pharmacological agents, this mode of block coincides with voltage-dependence (Cui et al., 2006, Newland and Cull-Candy, 1992). However, despite its negatively-charged sulphate moiety, PS block was not strongly voltage-dependent, with only 12% more block recorded at depolarised compared to hyperpolarised membrane potentials. Similar findings on the rate and extent of block have been reported by others in oocytes, HEK cells and rat cortical neurons (Akk et al., 2001, Eisenman et al., 2003, Majewska et al., 1988), strongly suggesting that PS does not act as an open-channel blocker and that its binding site is most likely located outside the channel pore. Also, the sulphate moiety of PS does not seem to be essential for GABA_A_R inhibition (Park-Chung et al., 1999, Seljeset et al., 2015) and would explain why the binding of PS is relatively unaffected by the membrane electric field.

The profile of block by PS has some similarity to that of PTX at low GABA concentrations (Eisenman et al., 2003). Early studies indicated that PTX displaced PS from rat brain membranes (Majewska et al., 1990), and PS competitively inhibited the binding of the PTX-like blocker, t-butylbicyclophosphorothionate (TBPS) in rat synaptosomes (Majewska and Schwartz, 1987). Radioligand displacement is often interpreted as compounds competing for a common binding site, but for allosteric proteins like the GABA_A_R, this need not be the only interpretation. Furthermore, mutating the 2’ channel residue in the GABA_A_R α and/or β subunits led to the identification of a possible binding site for PTX in the channel pore (Xu et al., 1995, Zhang et al., 1994), which is supported by the crystal structure of GluCl bound to PTX at this site (Hibbs and Gouaux, 2011). However, a single channel site for PS and PTX seems unlikely as competition experiments with α1β2γ2L revealed no interaction and thus inhibitory effects on GABA currents that were additive. Moreover, block by PS and PTX is different at high GABA concentrations, with PTX having little or no effect on steady-state currents (Eisenman et al., 2003), contrasting with the marked attenuation of steady-state currents by PS at high GABA concentrations. This strengthens the hypothesis that the mechanism of block by PS and PTX are distinct and likely mediated by separate binding sites.

With regard to accessing the binding site, unlike the potentiating neurosteroids which can partition into the plasma membrane and exert their effect from the cytosol (Akk et al., 2009, Akk et al., 2007), intracellular PS was ineffective and only reached its binding site from the external side of the membrane. This result also suggests PS has a defined binding site rather than affecting the receptor protein in a non-specific manner. This result has some similarity with another target of PS, the TRPM3 channel, which can only be activated by extracellular, but not intracellular PS (Wagner et al., 2008).

The GABA_A_R with a distinctive lack of PS sensitivity (below 100 μM) was the ρ1 receptor. This contrasts with the potentiating neurosteroids, which are positive and negative allosteric modulators at ρ1, though the concentrations required to achieve this are higher than at heteromeric receptors (>1 μM) (Morris et al., 1999). In determining which receptor domains were crucial for PS inhibition, it became clear that several subunits could be involved. The sensitivity of α1β2 to PS indicates that binding does not depend solely on the γ2L subunit. Furthermore, inhibition of PB-mediated currents confirmed the presence of a binding site for PS on the β3 subunit, which does not exclude potential sites on α1-6 and γ2L subunits.

**Fig. 9:**
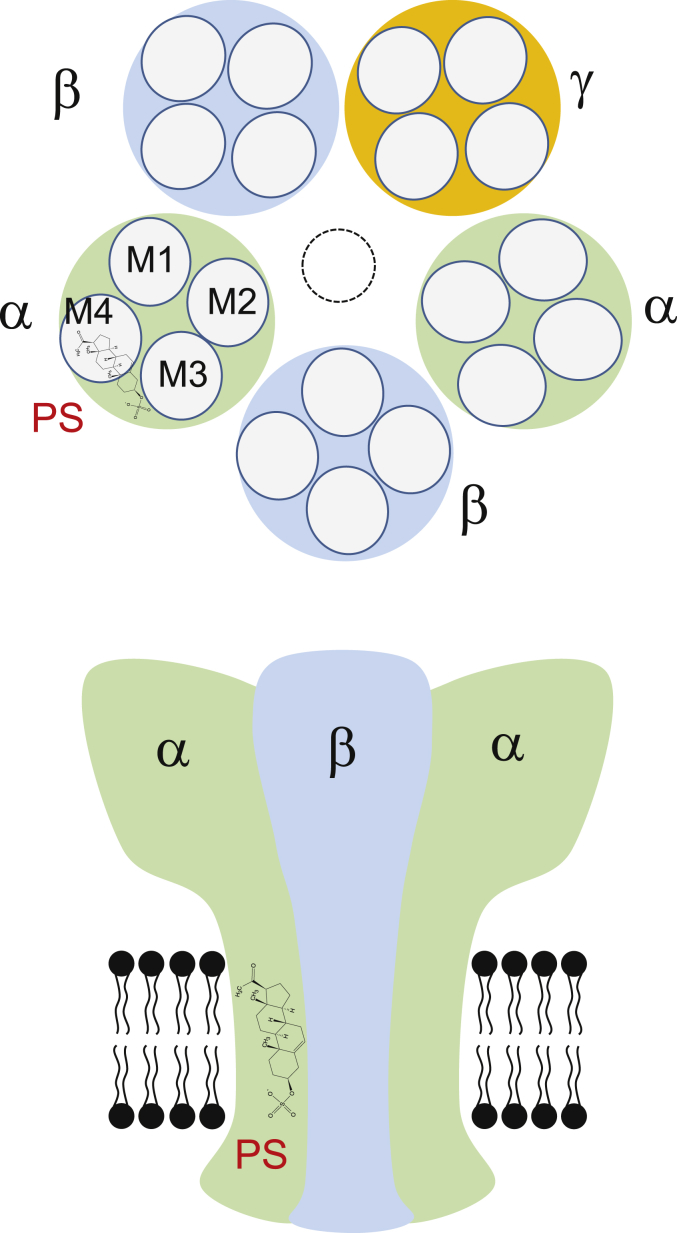
Schematic diagram of a PS binding site. Upper panel depicts a plan view of a hetero-pentameric GABA_A_R containing αβγ subunits at the level of the TMD. Subunits are labelled and a proposed binding location for PS is shown on the α subunit near the α-helices representing M3 and M4. Lower panel shows a side view of the same receptor schematic in the membrane with PS occupying a site which does not involve lipid partitioning or the channel pore, in keeping with the poor voltage sensitivity for inhibition.

Various chimeras between ρ1 and α1, β2 or γ2 subunits were examined in searching for the critical domains for PS inhibition. At first it seemed surprising that chimeras with ECD-TMD drawn from α, β or γ subunits (ECD) with ρ1 (TMD), or vice-versa, retained some sensitivity to PS. However, for the chimeric receptor complex containing the TMD of ρ1 and ECD of α1, β2 and γ2 (α1-222-ρ1 + β2-218-ρ1 + γ2-235-ρ1) it appears likely that only the constitutive current, rather than the GABA current, was inhibited by PS implying that residues in the TMDs of the α1, β2 and/or γ2 subunits are necessary for PS inhibition of the GABA-activated receptor. In support of this, chimeras containing the ECD of ρ1 and the TMDs of α1 or γ2 (ρ1-260-α1/γ2) were sensitive to PS, even those including only M4 of the α1 subunit. Taken together, this suggests that PS is likely to bind to the TMDs of α, β and γ subunits (Fig. 9). It is also likely that the homomeric wild-type ρ1 receptor fails to support profound PS inhibition because of an absent signalling mechanism, since mutating the channel 2′ residue to that found in α1 can render ρ1 more sensitive to PS. The residues necessary for this allosteric mechanism are likely to be present in α1, β2 and γ2 subunits, and absent in the wild-type ρ1 receptor. This accumulated evidence for the importance of the TMDs in PS sensitivity is emphasised by recent work on a GLIC-GABA_A_Rα1 subunit chimera (Laverty et al., 2017), in which a PS binding site is tentatively located using X-ray crystallography to a TMD site involving a ‘longitudinal groove’ between M3 and M4 of the α1 subunit. This does not preclude additional binding in the TMD of other GABA_A_R subunits such as β and γ2.

Earlier studies of single channel recordings (Akk et al., 2001) revealed that the α1 subunit 2′ mutation, α1^V256S^, in αβγ receptors reduced the apparent association rate of PS 30-fold and prevented the reduction in GABA channel cluster duration by PS. Homologous mutations in the β2 and γ2L subunits had no such effect. Our study showed that the PS IC_50_ for GABA steady-state currents was decreased by 89-fold when the α1 subunit is mutated at 2′ for α1^V256S^β2γ2L receptors. Conversely, for peak GABA current inhibition, the PS IC_50_ is decreased. Reduced or abolished inhibitory effects of PS on GABA currents have been reported for α1^V256S^β2γ2L and α1β2^A252S^γ2L receptors (Wang et al., 2006, 2007). This supports the notion that mutating either the α1 or β2/3 subunits at the 2′ position reduces the potency and efficacy of PS. However, taking all data into consideration, and with the low voltage-dependence of PS, it is more likely that the 2’ mutation alters an allosteric mechanism and interferes with signal transduction rather than directly affects the binding of PS.

Interestingly, α1^V256S^, but not β2^A252S^, eliminates GABA_A_R inhibition by the 3β-hydroxypregnane steroids (Seljeset et al., 2015, Wang et al., 2002, 2007). These are diastereomers of the potentiating 3α-hydroxypregnane steroids, but are similar to the sulphated neurosteroids in that they non-competitively inhibit the GABA_A_R in an activity- or state-dependent manner. In the study by Wang et al. (2007), desensitisation kinetics were characterised by determining a ratio between peak and steady-state currents (I_P_/I_SS_). In wild-type receptors, PS increased the ratio in a concentration-dependent manner, but this remained unchanged when the 2’ mutation was introduced to the α1 or β2 subunit.

This suggests that PS promotes desensitisation of wild-type receptors, an effect that is removed by the mutations. Notably, the block by the 3β-hydroxypregnane steroids did not cause a concentration-dependent increase in the I_P_/I_SS_ ratio in wild-type or mutant receptors, suggesting that the mechanism of block by sulphated steroids and 3β-hydroxypregnane steroid is not common (Wang et al., 2007). From this we deduce that the 2’ residue is unlikely to be a common binding site for either group of steroids, and corroborates the hypothesis that this is likely to be a residue important for allosteric signalling (Seljeset et al., 2015).

What do these data accrued from recombinant receptors mean for the actions of PS in the nervous system? Although it is difficult to be precise, by PS promoting a desensitised state, we might assume that low frequency inhibitory synaptic potentials (IPSPs) would be relatively unaffected by PS in the absence of overt receptor desensitisation. However, high frequency release, with summation of IPSPs and desensitisation, may show some degree of attenuation by PS. Moreover, given that extrasynaptic GABA_A_Rs can be desensitised by persistent exposure to low concentrations of GABA (Mortensen et al., 2010, Bright et al., 2011) we may expect some moderation by PS of the level of tonic inhibition.

In conclusion, PS exhibits minimal GABA_A_R subtype selectivity, and the extent to which this negative allosteric modulator blocks the GABA_A_R increases with receptor activation that is likely to promote the desensitised state. The domains that are pivotal to PS inhibition are centred on the subunit TMDs, which concurs with recent high resolution x-crystallographic data that suggests an involvement of this domain in the GABA_A_R for PS inhibition.
